# Idiopathic pulmonary fibrosis: the radiologist’s role in making the diagnosis

**DOI:** 10.1259/bjr.20181003

**Published:** 2019-05-14

**Authors:** Michael P. Mohning, John Caleb Richards, Tristan J. Huie

**Affiliations:** 1 National Jewish Health, Denver, Colorado, USA

## Abstract

Radiologists have a critical role in the evaluation and diagnosis of suspected idiopathic pulmonary fibrosis (IPF). Accurate pattern identification on imaging is key in the multidisciplinary diagnostic process and frequently obviates the need for a surgical lung biopsy. In this review, we describe the clinical and imaging features of IPF in the context of recently revised international guidelines; contrast findings in other diseases that may inform differential diagnosis of fibrotic lung disease; and highlight common complications associated with pulmonary fibrosis.

## Introduction

Idiopathic pulmonary fibrosis (IPF) is a debilitating and ultimately fatal scarring lung disease. It is the most common form of interstitial lung disease (ILD) and is defined as a chronic, progressive fibrosing interstitial pneumonia of unknown cause with a usual interstitial pneumonia (UIP) pattern.^1^ The diagnosis of IPF requires a combination of medical history, laboratory assessment, radiology, and sometimes pathology. An accurate diagnosis is critical given the poor prognosis of the disease, the availability of antifibrotic therapies that slow the progressive course of IPF,^2,3^ and the fact that immunosuppressive drugs, which are used to treat other forms of ILD, are harmful for patients with IPF.^4^

Radiologists have a key role in the diagnosis and management of IPF (see Table 1). In 2011, through a collaborative effort between the American Thoracic Society, European Respiratory Society, Japanese Respiratory Society, and Latin American Thoracic Association, evidence-based guidelines for the diagnosis of IPF, including an imaging classification scheme, were introduced.^5^ Based on new data generated since 2011, two new statements were introduced in 2018—a clinical practice guideline from the same international societies^1^ and a white paper from the Fleischner Society.^6^ This review will provide a brief clinical review of IPF, address the radiologist’s role in the context of recent revisions to the diagnostic approach, and discuss imaging findings that help contribute to the multidisciplinary diagnostic process.

**Table 1.** Role of radiologist in the care of patients with IPF

Identify pattern of involvement if UIP considered: UIP Probable UIP Indeterminate for UIP Suggestive of an alternative diagnosis
Find clues to suggest an underlying etiology
Identify potential comorbidities or complications
Participate in a multidisciplinary discussion
Monitor progression of diseases

IPF, idiopathic pulmonary fibrosis; UIP, usual interstitial pneumonia.

## Epidemiology

IPF primarily affects older patients. The mean age at time of diagnosis is 65 years, and men are more commonly afflicted than females.^7^ Pulmonary fibrosis in a patient younger than 50 years old is rarely due to IPF—connective tissue disease, exposure-related disease, or rarely idiopathic non-specific interstitial pneumonia (NSIP) should be suspected. The annual incidence of IPF in the USA has been estimated to be between 6.8 and 16.3 per 100,000 people.^8^ A history of cigarette smoking is a risk factor, with about two-thirds of IPF patients being former or current smokers.^5,9^ There are a number of genetic mutations that place patients at risk for IPF. These include telomerase mutations, surfactant protein C mutations, and the Muc5B single nucleotide polymorphisms. Pulmonary fibrosis may be familial in up to 20% of cases.^10^

## Presentation and natural history

IPF typically presents with progressive dyspnoea on exertion with a chronic, non-productive cough. Patients may present months or even years after their symptoms develop. On examination, bibasilar "Velcro" crackles are present on inspiration,^11^ and the patient may have digital clubbing. The exam should seek extrapulmonary signs of a systemic disease associated with the development of ILD.^1,6^ Pulmonary function testing typically reveals restriction with decreased lung volume and a reduced diffusion capacity.^7^ Common comorbid conditions include pulmonary hypertension, emphysema, obstructive sleep apnoea, coronary artery disease, and gastroesophageal reflux.^12^ The clinical course of IPF is heterogeneous: most patients experience a gradual decline in lung function, whereas others show a rapid deterioration in the 6–12 months after diagnosis.^13^ Some patients experience acute respiratory deteriorations, known as acute exacerbations, which are often fatal.^14^ Overall, IPF has a very poor prognosis, with an average post-diagnosis survival of 3–4 years.^15^

## Diagnostic approach

There are several other forms of ILD that need to be considered when making a differential diagnosis of IPF (Table 2). Non-specific interstitial pneumonia, commonly associated with connective tissue disease-associated ILD (CTD-ILD) or rarely idiopathic, and chronic hypersensitivity pneumonitis (HP) are the most commonly considered alternative diagnoses. Other potential etiologies that should be considered include drug reaction, radiation-induced fibrosis, sarcoidosis, and occupational exposures (*e.g.* asbestosis). The clinician must evaluate the context in which the ILD developed, excluding an underlying cause of ILD, based on the patient’s clinical presentation, a detailed history including exposures (environmental antigens, asbestos, medications, etc.), and laboratory testing.^1,6^ High-resolution CT (HRCT) is performed to determine the pattern of lung abnormality. The context and pattern of injury are used to determine the diagnosis, ideally in the context of a multidisciplinary discussion (MDD) that involves clinicians, radiologists, and pathologists.^1,6^

**Table 2.** Differential diagnoses in patients with suspected IPF

Connective tissue disease-associated ILD
Hypersensitivity pneumonitis
Occupational lung disease
Drug-induced lung disease
NSIP
Sarcoidosis

ILD, interstitial lung disease; IPF, idiopathic pulmonary fibrosis;NSIP, non-specific interstitial pneumonia.

## Laboratory testing

Laboratory testing is aimed at identifying potential etiologies for the patient’s ILD, primarily to assess for the presence of an autoimmune disease or if there is concern for HP. This serologic testing often includes anti-nuclear antibody, rheumatoid factor, anti-cyclic citrullinated peptide, scleroderma antibodies (SCL-70), and an anti-synthetase autoantibody panel.^1^ HP antigen panels are not sensitive enough to rule out HP, but may be beneficial in suspected cases. Analysis of bronchoalveolar lavage (BAL) fluid is used to assess cell counts and differentials. BAL from patients with IPF typically does not show high levels of lymphocytes, as is frequently seen in HP, sarcoidosis, or CTD-ILDs.^16^ The latest international diagnostic guidelines do not recommend cellular analysis of BAL fluid in patients who are clinically suspected of having IPF and have an HRCT pattern of UIP, but recommend that BAL fluid be analyzed in patients who are clinically suspected of having IPF but have other patterns on HRCT.^1^

## Pathology

**Figure 1.**
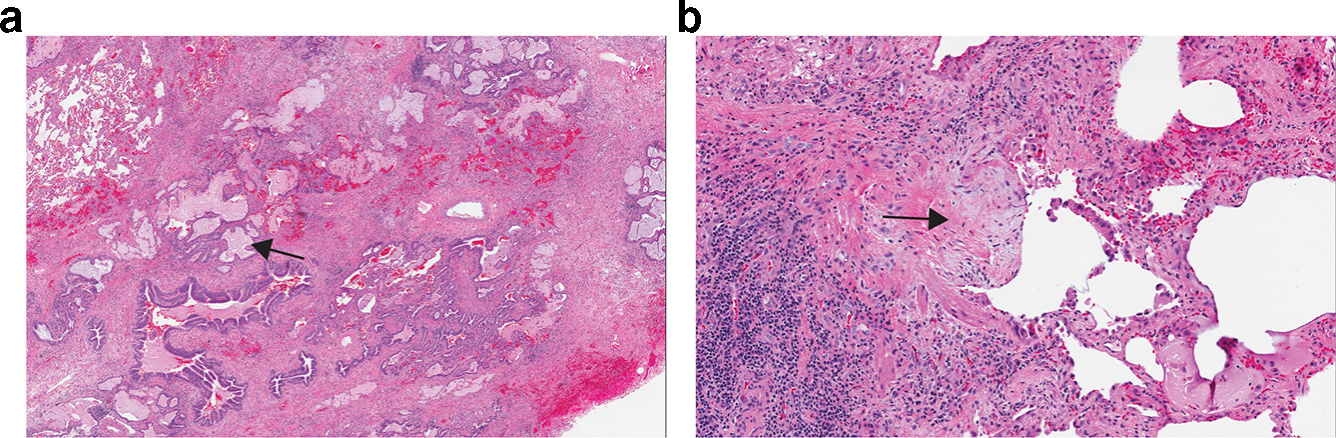
(a) Lower power image of a surgical lung biopsy specimen demonstrating a UIP pattern with honeycombing (arrow) and scattered fibrosis. (b) Higher power image of the biopsy showing a fibroblastic focus (arrow). UIP, usual interstitial pneumonia.

A surgical lung biopsy may be required to make a diagnosis of IPF when medical history and imaging are not definitive. Pathology patterns may be classified as UIP, probable UIP, indeterminate for UIP, or suggestive of an alternative diagnosis.^1^ The hallmarks of a UIP pathologic pattern include temporal and geographic heterogeneity of fibrosis intermixed with normal lung; the presence of fibroblastic foci; and often microscopic honeycomb changes (Figure 1). The disease is most marked in peripheral and subpleural areas of the lung. There should be an absence of features that suggest an alternative cause, such as signs of injury, marked inflammation, granulomas, or an airway-focused distribution. A histological UIP pattern can be seen in other ILDs such as chronic HP,^17^ CTD-ILDs,^18,19^ and drug toxicity,^20^ but when a UIP pattern occurs in a patient with no identifiable cause for ILD, the diagnosis is IPF. Current international guidelines suggest that to obtain a definite diagnosis of IPF, a surgical lung biopsy needs to be conducted in patients who do not have a typical UIP pattern on HRCT, but acknowledge that surgical lung biopsy is not indicated in patients at high risk of complications.^1^ For an individual patient, the benefits of obtaining a more confident diagnosis need to be weighed against the risks of them undergoing surgical lung biopsy.^21^ The high proportion of UIP on pathology among patients with probable UIP on imaging resulted in the Fleischner Society recommendation that in the appropriate clinical context, patients with a probable UIP pattern on CT warrant a diagnosis of IPF without undergoing surgical lung biopsy.^6^ Our practice is more consistent with these guidelines, with a preference for not subjecting patients with a probable UIP pattern to biopsy, given the rarity of identifying a non-UIP pattern or pathologic features that suggest an alternative diagnosis. The latest international diagnostic guidelines made no recommendation for or against transbronchial biopsy or cryobiopsy in the diagnosis of IPF.^1^

## Radiology

HRCT of the chest is superior to other imaging modalities in the detection and assessment of the extent of ILD due to its superior contrast resolution.^22^ Current volumetric multidetector HRCT protocols optimize radiation dose reduction while maintaining image quality,^23^and allow multiplanar reformatting for a better assessment of pattern and distribution of abnormality (particularly craniocaudal distribution). It also improves assessment of progression of fibrosis on follow-up imaging. It also better depicts comorbidities such as lung cancer or infection. Expiratory imaging should be strongly considered to evaluate air trapping, which may suggest an alternative diagnosis such as HP. Prone imaging may help if the fibrosis is mild or there is dependent density obscuring detail on supine imaging.

### Updated classification scheme for UIP

**Figure 2.**
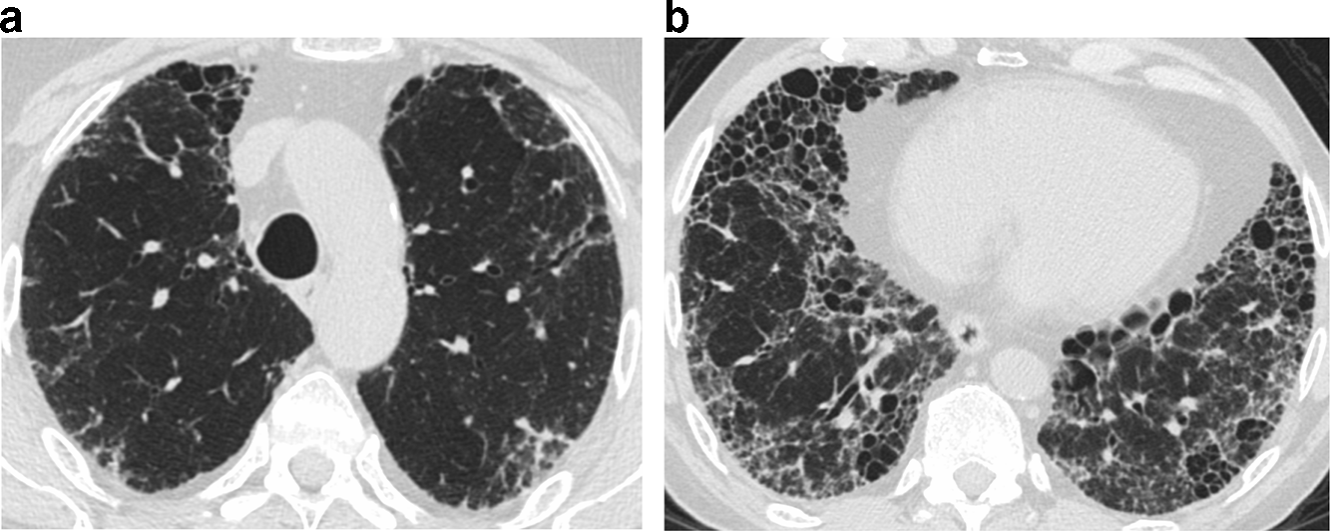
Typical UIP on HRCT. Axial images at cranial (a) and caudal (b) level show subpleural fibrosis with reticulation, traction bronchiectasis, and honeycombing. The extent of fibrosis is worse at the more caudal level, consistent with lower lung predominance of fibrosis. The basal and subpleural distribution and associated honeycombing are requisite for typical UIP. HRCT, high-resolution CT; UIP, usual interstitial pneumonia.

**Figure 3.**
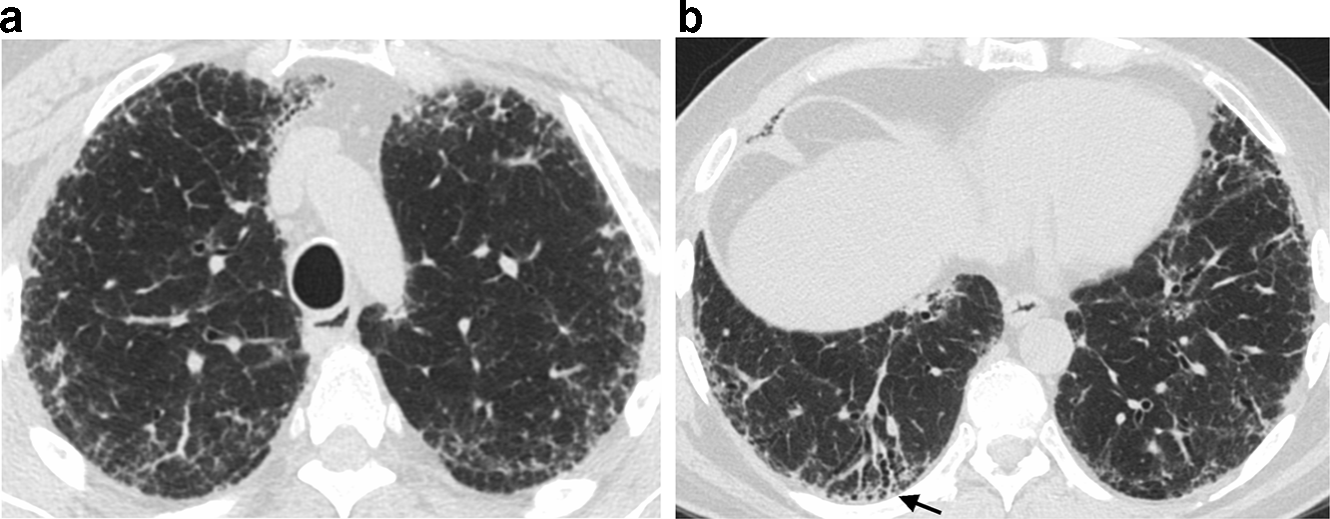
Probable UIP on HRCT. Subpleural reticular upper abnormality in the upper lungs (a) with increasing fibrosis and traction bronchiectasis (arrow) at a more caudal (b) level. The pattern appears similar to typical UIP, but lacks honeycombing, and thus is labelled probable UIP. HRCT, high-resolution CT; UIP, usual interstitial pneumonia.

**Figure 4.**
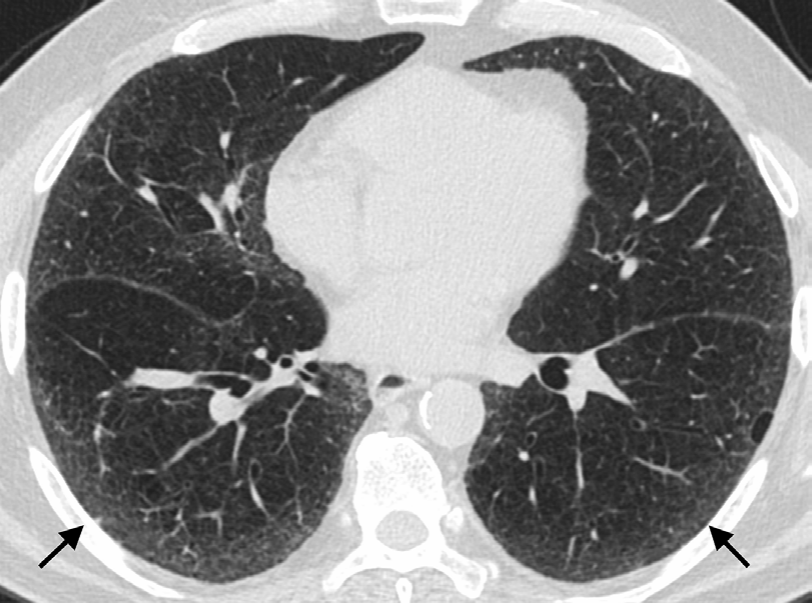
Indeterminate for UIP on HRCT. Mild interstitial abnormality characterized by ground glass (arrows) opacity with a subpleural predominant distribution. This pattern is labelled indeterminate for UIP because of the disproportionate ground glass opacity and overall mild extent. Histological UIP was present on surgical lung biopsy. HRCT, high-resolution CT; UIP, usual interstitial pneumonia.

The new international guidelines expanded the HRCT classification for UIP to four categories: typical UIP, probable UIP, indeterminate for UIP, or suggestive of an alternative diagnosis. A typical UIP pattern shows a subpleural and basal distribution of fibrosis characterized by honeycombing, with/without peripheral traction bronchiectasis or bronchiolectasis, and the absence of findings that suggest a diagnosis other than IPF (Figure 2). A probable UIP pattern lacks honeycombing but otherwise shows the features of a typical UIP pattern (Figure 3). Under the 2011 guidelines, this pattern would have been labeled as “possible UIP.” However, subsequent studies demonstrated that patients with this pattern who undergo surgical lung biopsy are likely to have histologic UIP.^24,25^ The radiologic pattern of “indeterminate for UIP” is characterized by peripheral and basal predominant reticulation without features that suggest a specific etiology. The reticular abnormality is often mild and there may be associated ground glass opacity (Figure 4). Although histologic UIP is possible, the findings in this HRCT pattern are not sufficient to confidently choose a diagnosis.

**Figure 5.**
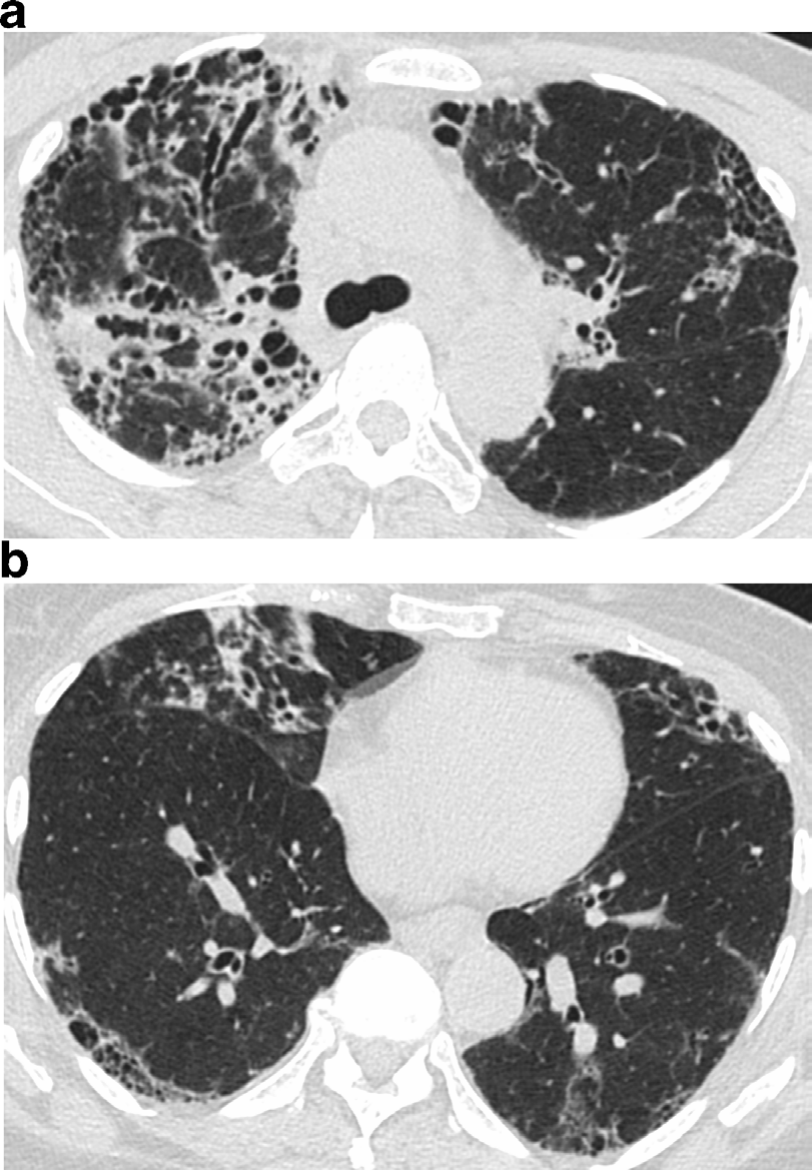
HRCT features suggestive of an alternative diagnosis. There is worse fibrosis on the cranial image (a) compared to a more caudal level (b), with traction bronchiectasis and distortion showing subpleural and bronchovascular distribution. The upper lung predominance suggests a non-IPF diagnosis. This patient was diagnosed with sarcoidosis. HRCT, high-resolution CT; IPF, idiopathic pulmonary fibrosis.

The fourth category includes features that suggest an alternative diagnosis to IPF (Figure 5). Findings that suggest a diagnosis other than IPF are based on CT features (cysts, marked mosaic attenuation, extensive ground glass opacities, profuse micronodules, centrilobular nodularity, nodules, or areas of consolidation), distribution of disease (peribronchovascular, perilymphatic, or upper or mid-lung zone distribution), or additional features that suggest an underlying systemic disease (*e.g.* pleural plaques found in asbestosis; features associated with autoimmune disease such as dilated esophagus, articular involvement or pericardial or pleural disease) (Table 3). This pattern was previously labeled as “inconsistent with UIP”^5^ but many patients with this HRCT pattern have a histologic pattern of UIP. In a group of patients enrolled in a clinical trial for IPF, 31% of patients had an imaging pattern that was inconsistent with UIP, but 97% of these patients showed a definite or probable pattern of UIP on pathology. The most common reasons these scans were interpreted as suggesting an alternative diagnosis were diffuse mosaic attenuation, upper or mid-lung zone predominance, and extensive ground-glass abnormalities.^26^

**Table 3.** Radiographic patterns of IPF (Reproduced with permission from Raghu et al. 2018)

HRCT patterns			
UIP	Probable UIP	Indeterminate for UIP	Alternative diagnosis
Subpleural and basal predominant; distribution is often heterogeneous^a^	Subpleural and basal predominant; distribution is often heterogeneous	Subpleural and basal predominant	Findings suggestive of another diagnosis, including:
Honeycombing with or without peripheral traction bronchiectasis or bronchiolectasis^b^	Reticular pattern with peripheral traction bronchiectasis or bronchiolectasis	Subtle reticulation; may have mild GGO or distortion (“early UIP pattern”)	CT features: Cysts Marked mosaic attenuation Predominant GGO Profuse micronodules Centrilobular nodules Nodules Consolidation
	May have mild GGO	CT features and/or distribution of lung fibrosis that do not suggest any specific etiology (“truly indeterminate for UIP”)	Predominant distribution Peribronchovascular Perilymphatic Upper or mid-lung
			Other: Pleural plaques (consider asbestosis) Dilated esophagus (consider CTD) Distal clavicular erosions (consider RA) Extensive lymph node enlargement (consider other etiologies) Pleural effusions, pleural thickening (consider CTD/drugs)

CTD, connective tissue disease; GGO, ground-glass opacity; HRCT, high-resolution computed tomography; IPF, idiopathic pulmonary fibrosis; RA, rheumatoid arthritis; UIP, usual interstitial pneumonia.

Reprinted with permission of the American Thoracic Society.Copyright © 2018 American Thoracic Society. Raghu G, et al. 2018 Diagnosis of idiopathic pulmonary fibrosis. An Official ATS/ERS/JRS/ALAT clinical practice guideline. American Journal of Respiratory and Critical Care Medicine 198:e44-e68. The American Journal of Respiratory and Critical Care Medicine is an official journal of the American Thoracic Society.

a Variants of distribution: occasionally diffuse, may be asymmetrical.

b Superimposed CT features: mild GGO, reticular pattern, pulmonary ossification.

### Features of fibrosis

**Figure 6.**
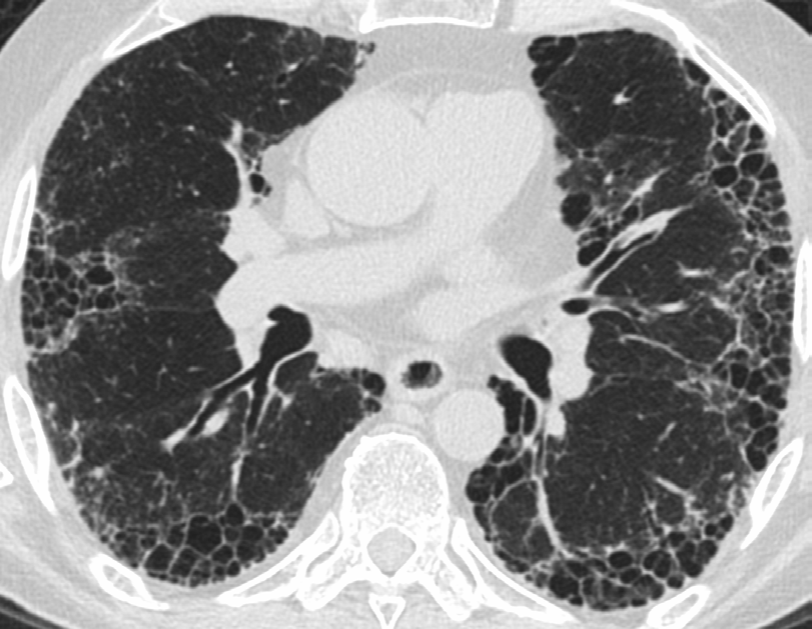
Multiple foci of honeycombing in UIP. Note the uniform cysts with a stacked appearance located in the subpleural lung, characteristic of honeycombing. UIP, usual interstitial pneumonia.

Knowing the features of fibrosis can help in placing an image into a specific pattern. Honeycombing on CT is defined as clustered cystic air spaces in a subpleural location, typically of comparable diameters of the order of 3–10 mm, but occasionally as large as 2.5 cm.^27^ (Figure 6). Importantly, while the term honeycombing is used both radiologically and histologically, the definitions differ and should not be used interchangeably. Histologic honeycombing is described as “destroyed and fibrotic lung tissue with numerous cystic air spaces with thick fibrous walls,” with some researchers describing a size of 1–2 mm.^28–30^ Studies have shown only moderate interobserver agreement in the identification of honeycombing on HRCT even among expert radiologists.^31,32^ The discrepancy may be related to disagreement on the definition of honeycombing itself, as well as mimics such as traction bronchiectasis, cystic air spaces, and paraseptal emphysema. Contiguous images are often helpful in distinguishing honeycombing from its mimics.

Reticulation is defined as linear opacity related to interlobular septal thickening, intralobular lines, or the cystic walls of honeycombing (though reticular pattern and honeycombing should not be considered synonymous).^27^ The extension of reticular abnormality into the upper lungs has been shown to be an independent predictor of UIP at histology, and the combination of lower lung honeycombing and upper lung irregular lines gives a specificity of 81% and positive predictive value of 85% for histologic UIP.^33^ Traction bronchiectasis or bronchiolectasis is caused by the irreversible dilation of bronchi and bronchioles from adjacent fibrotic and distorted lung architecture.^27^ Ground glass opacity refers to increased lung attenuation which does not obscure underlying bronchovascular structures. Ground glass opacity is allowed in a UIP pattern if present in areas of fibrotic parenchyma. If in areas of nonfibrotic parenchyma, alternate etiologies should be considered.

### Differential diagnosis

**Figure 7.**
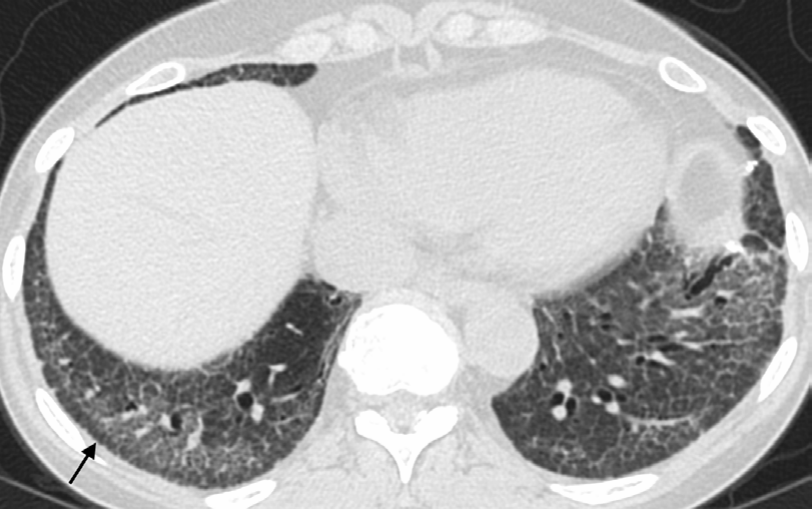
NSIP pattern with relatively homogeneous ground glass opacity, fine reticulation, and traction bronchiectasis. Subpleural sparing (arrow) is unusual for UIP but more specific for NSIP. NSIP, non-specific interstitial pneumonia; UIP, usual interstitial pneumonia.

When imaging does not show a definite UIP pattern, it is important for the radiologist to be aware of imaging features that may suggest a diagnosis other than IPF. A radiological pattern of NSIP is most commonly associated with CTD, but can also be caused by chronic HP, drugs, or occupational exposure, or be idiopathic.^34^ On HRCT, NSIP most commonly shows symmetric, lower lung distribution of abnormality, with ground glass opacity being the salient feature. Reticulation and traction bronchiectasis may be present depending on the degree of fibrosis, but honeycombing is less common.^35^ Subpleural sparing is a suggestive feature that helps distinguish NSIP from other radiological patterns (Figure 7).^36^ However, the ability of HRCT to predict histologic NSIP is not nearly as accurate as the ability of HRCT to predict histologic UIP. In one study, only 18 of 44 patients who were called definite or probable NSIP on HRCT actually had histologic NSIP, while the remainder had histologic UIP.^37^

**Figure 8.**
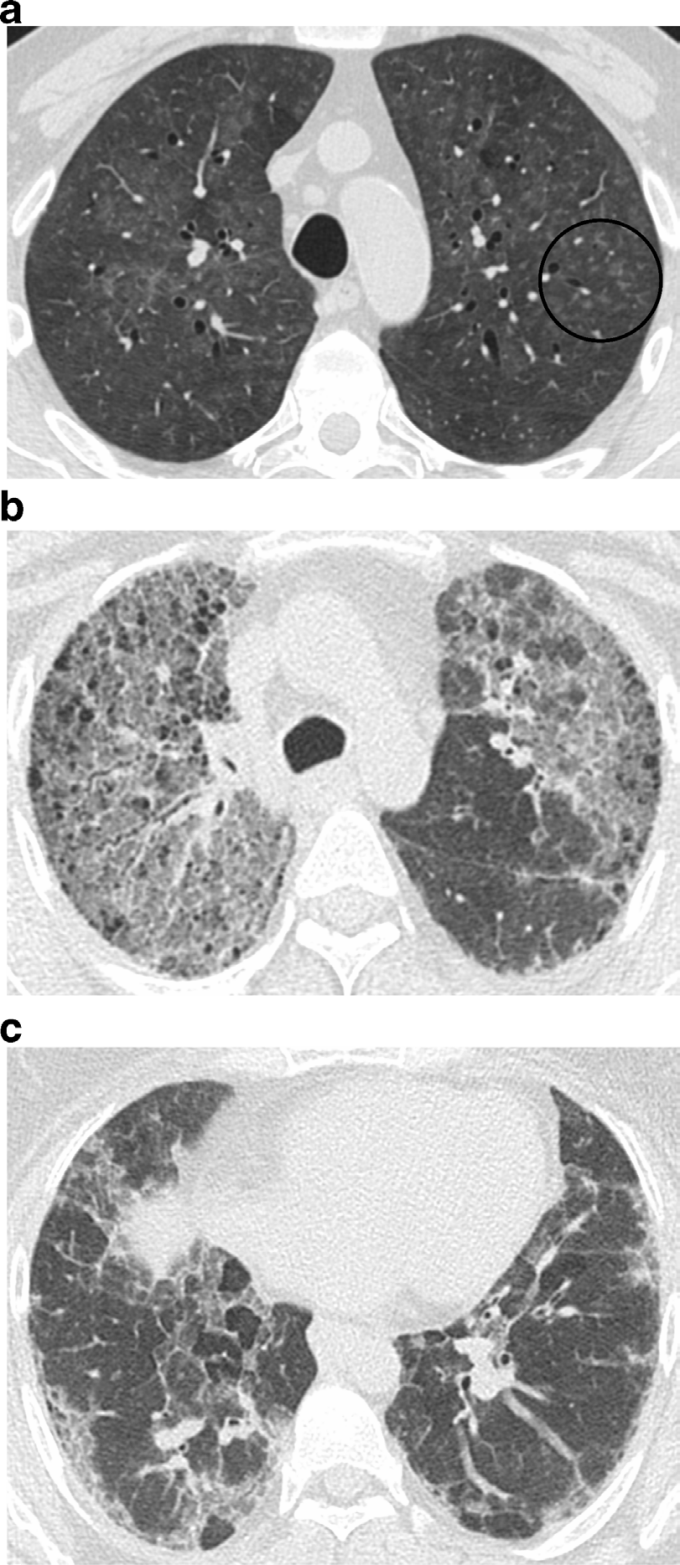
Two patients with hypersensitivity pneumonitis. The first patient shows a mosaic pattern with patchy and centrilobular (circle) ground glass typical of HP, and very mild upper lung fibrosis manifesting as traction bronchiectasis (a). A second patient with more advanced fibrosis shows upper lung traction bronchiectasis, distortion, and ground glass (b) with much milder abnormality in the lower lungs (c). HP, hypersensitivity pneumonitis.

The HRCT findings that best distinguish chronic HP from other ILDs are centrilobular nodularity, ground glass opacity, upper lung predominance, and mosaic attenuation and air trapping which are often lobular in shape (Figure 8).^36,38–40^ While upper lung predominance has historically been described as a unique feature of HP, most patients with fibrotic HP actually have lower lung predominant fibrosis, and many meet criteria for a definite UIP pattern on HRCT.^36,40–42^ In a study of 72 patients with fibrotic HP, 57% had lower lung predominance of fibrosis and 37% met criteria for a UIP pattern.^42^

**Figure 9.**
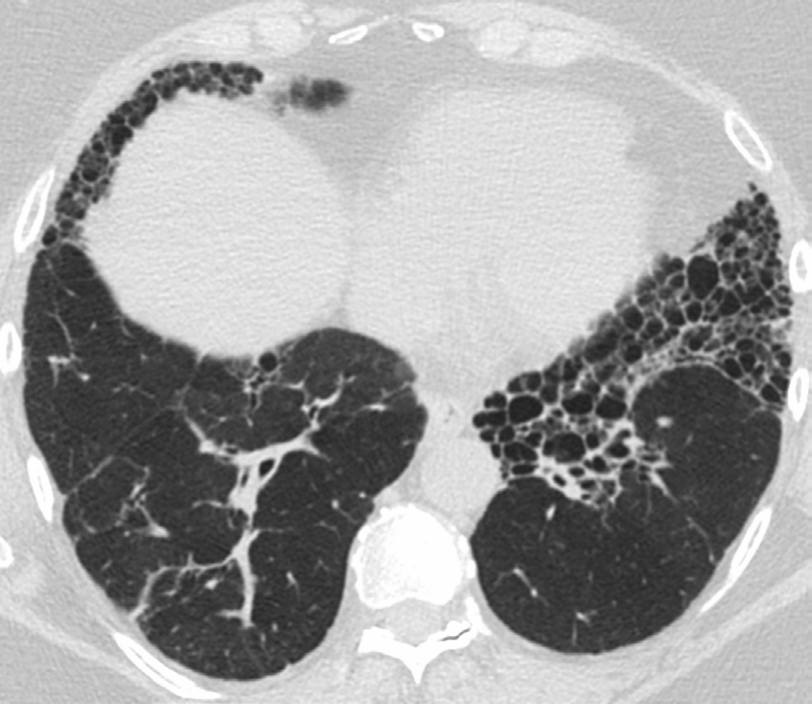
Exuberant honeycombing in a patient with UIP secondary to connective tissue disease. Note honeycombing is the predominant feature, accounting for over 70% of the fibrotic area. UIP, usual interstitial pneumonia.

**Figure 10.**
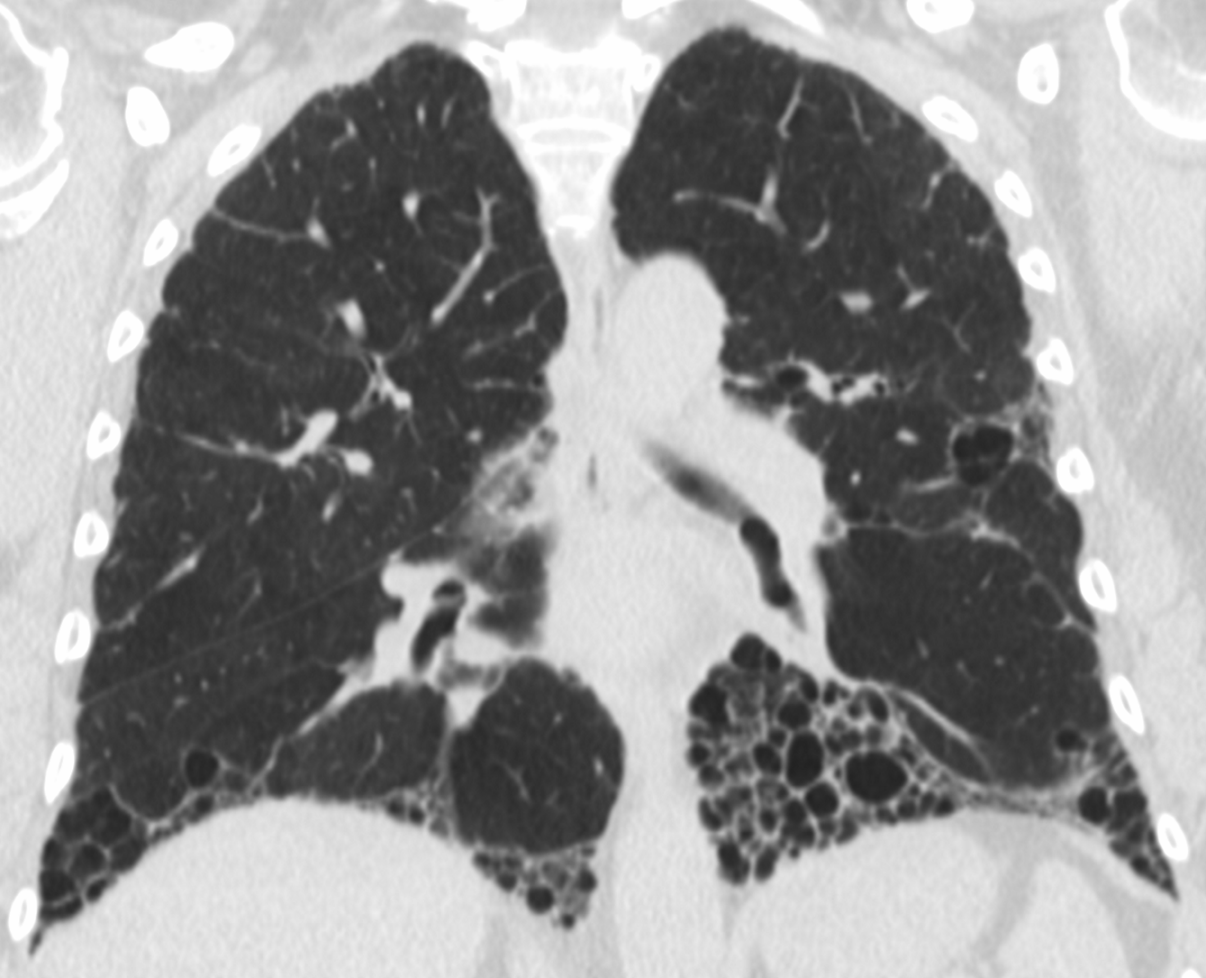
Straight edge sign in the same patient shown in Figure 9. This sign refers to a sharply demarcated transition between fibrotic and non-fibrotic parenchyma observed on coronal images.

Certain CT features have been suggested that may distinguish UIP associated with CTD from UIP associated with IPF. Chung and colleagues investigated the frequency of three of these features in patients with a radiographic pattern of UIP due to CTD (*n* = 63) or IPF (*n* = 133). They defined the “exuberant honeycombing” sign when honeycombing accounts for >70% of the fibrotic area of the lung; the “straight edge” sign as a sharp demarcation between fibrosis and normal lung in the craniocaudal plane; and the “anterior upper lobe” sign when fibrosis is concentrated in the anterior segment of the upper lobes relative to the other segments of the upper lobes, and fibrosis also occurs in the lung bases (Figures 9 and 10). These three CT signs occurred with greater frequency in patients with CTD (22.3–25.4%) than IPF (6.0–12.8%). The “straight edge” sign was the most specific (94%) and sensitive (25.4%) of the three.^43^

## Multidisciplinary review

Current guidelines recommend MDD of patients with suspected ILD with input from clinicians, radiologists, and pathologists to improve diagnostic accuracy.^1,6^ In a seminal study, pulmonologists, radiologists and pathologists evaluated data related to 58 cases of idiopathic interstitial pneumonias in a stepwise fashion and were asked to determine the diagnosis and diagnostic confidence at each step. Interobserver agreement and diagnostic confidence improved when these specialists discussed the cases together.^44^ The international IPF Project Consortium evaluated the diagnostic performance of over 400 physicians to evaluate cases of ILD and compared their performance to a panel of IPF experts. Participants were given clinical data and HRCT images and asked to give a differential diagnosis in order of likelihood and assign a prognosis. The study showed that pulmonologists at university-based centres made diagnoses of IPF with similar prognostic accuracy to a panel of IPF experts. Furthermore, regular MDD meeting attendance improved prognostic accuracy of experienced practitioners at non-university-based centres to levels achieved by IPF experts.^45^

An MDD based on clinical and imaging data should inform the decision on whether to perform a biopsy. The Fleischner Society recommendations argue that a working diagnosis of IPF may be made after MDD when diagnostic tissue is not available. Patients with a working diagnosis of IPF should be periodically re-evaluated to determine if their disease has evolved into a characteristic pattern (*e.g.* a UIP pattern on imaging).^6^ Disease progression might also support a working diagnosis of IPF; this was identified by the joint international statement as an area requiring further study.

The best format for MDD has not been determined. At many academic centres, these discussions occur in person; however, this is time consuming, generally not reimbursed, and it may be impractical to have all relevant specialties meet. Informal MDD by phone or email may be sufficient, but this has not been rigorously studied. To make the MDD most effective, it is important that relevant clinical information (*e.g.* on the patient’s clinical history, exposures and the results of serological tests) is shared with all parties in advance.^46^

## Complications

### Acute exacerbations

**Figure 11.**
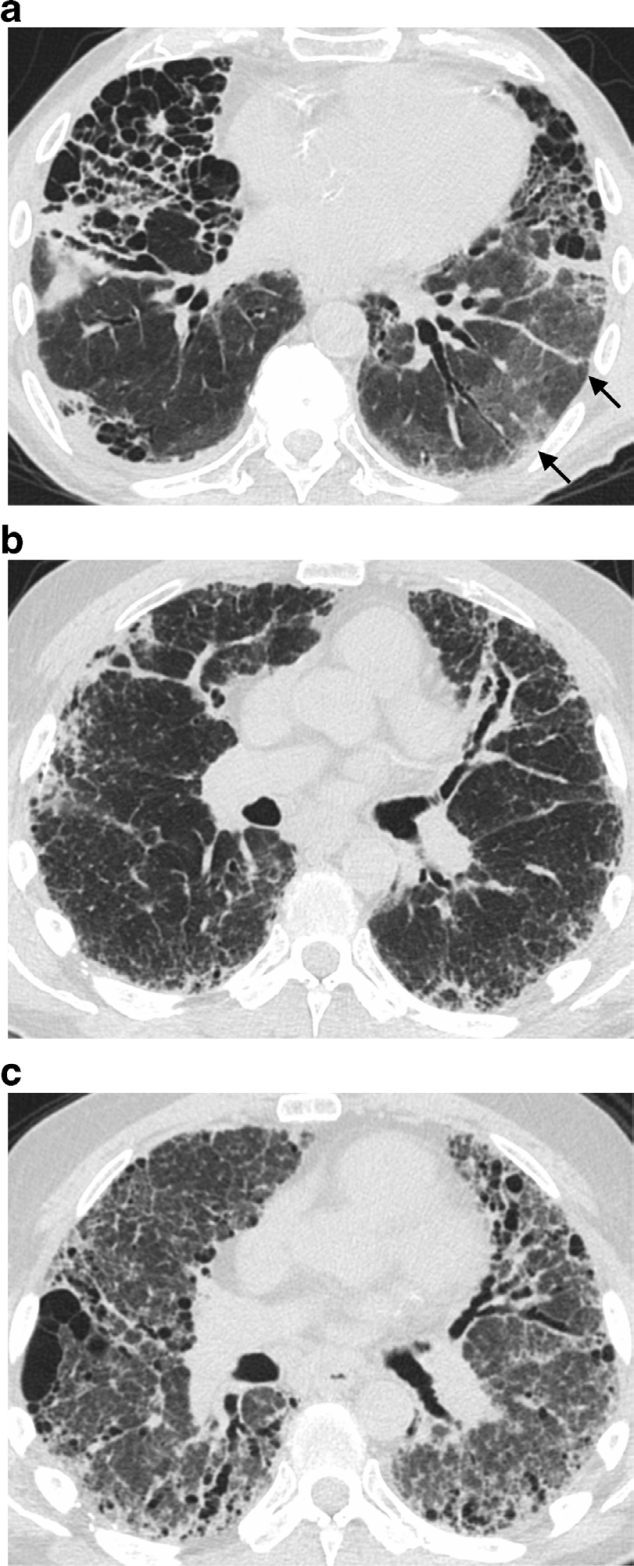
Acute exacerbation with peripheral (a) and diffuse (b, c) presentation. Note the peripheral ground glass is separate from fibrotic parenchyma (a). A separate patient with fibrosis shows clear development of diffuse ground glass opacity between images b and c, taken 6 months apart.

An acute exacerbation of IPF is defined as an acute, clinically significant respiratory deterioration characterized by evidence of new widespread alveolar abnormality.^14^ The development of bilateral ground glass opacities with or without consolidation in an acute setting (less than 1 month duration) is key, although edema or infection should be excluded as a primary contributor to the deterioration before attributing the opacities to an acute exacerbation.^14^ Ground glass opacities are typically diffuse on imaging, but peripheral and multifocal opacities may also be seen^47^ (Figure 11). When biopsied, diffuse alveolar damage is the typical histologic finding in an acute exacerbation, particularly in the organizing phase, with organizing pneumonia being the second most frequently seen pathologic pattern.^48,49^

### Lung cancer

**Figure 12.**
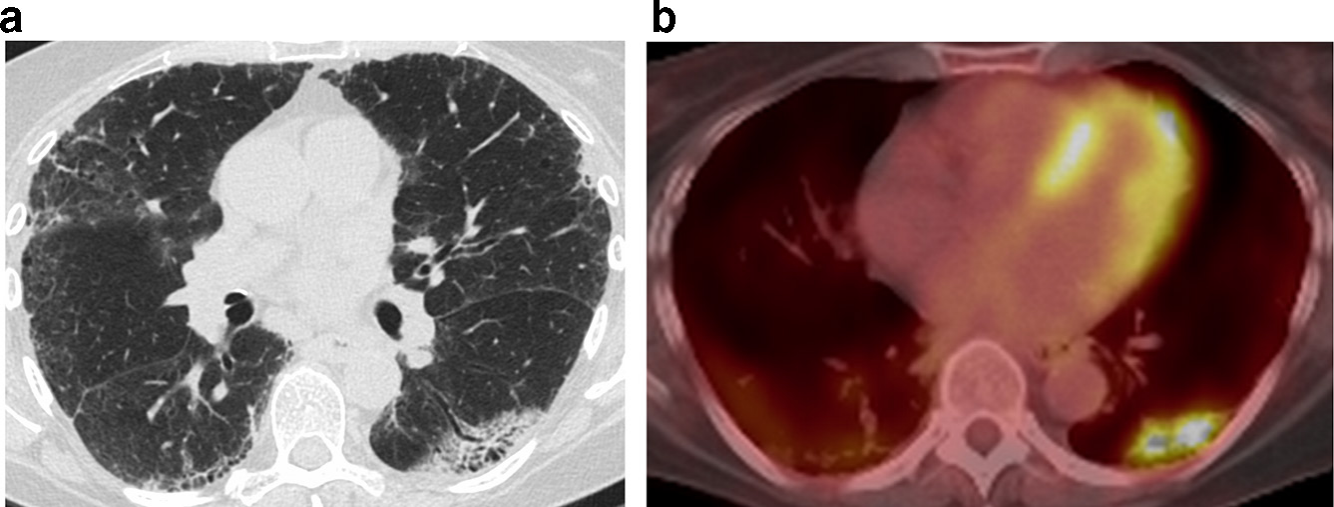
An irregular mass-like opacity is present in the left lower lobe in a region of fibrotic parenchyma (a). Note a mild background of subpleural fibrosis in the remaining lobes. Fluorodeoxyglucose avidity was apparent on positron emission tomography/CT (b). Histology revealed squamous cell carcinoma.

Patients with IPF, and particularly older males with a smoking history, have a significantly increased risk of lung cancer.^50,51^ When present in IPF patients, lung cancer frequently arises in the periphery of fibrotic areas, and shows a lower lung predominance similar to the zones of fibrosis, which may lead to a delay in diagnoses of these malignancies.^52–54^ One study evaluating lung cancer in patients with idiopathic interstitial fibrosis demonstrated that tumors occurred at the interface of fibrotic and normal lung parenchyma in 53% of cases.^54^ These tumors can vary in appearance and have been reported as typical nodules, both well-defined or lobulated, as well as areas of mass-like consolidations (Figure 12). Histologically these tumors are most commonly adenocarcinoma or squamous cell carcinoma.^51,54^ Because of the increased lung cancer risk in IPF patients, radiologists should pay special attention to these peripheral fibrotic areas for any new or progressive abnormalities.^54^

### Pulmonary hypertension

**Figure 13.**
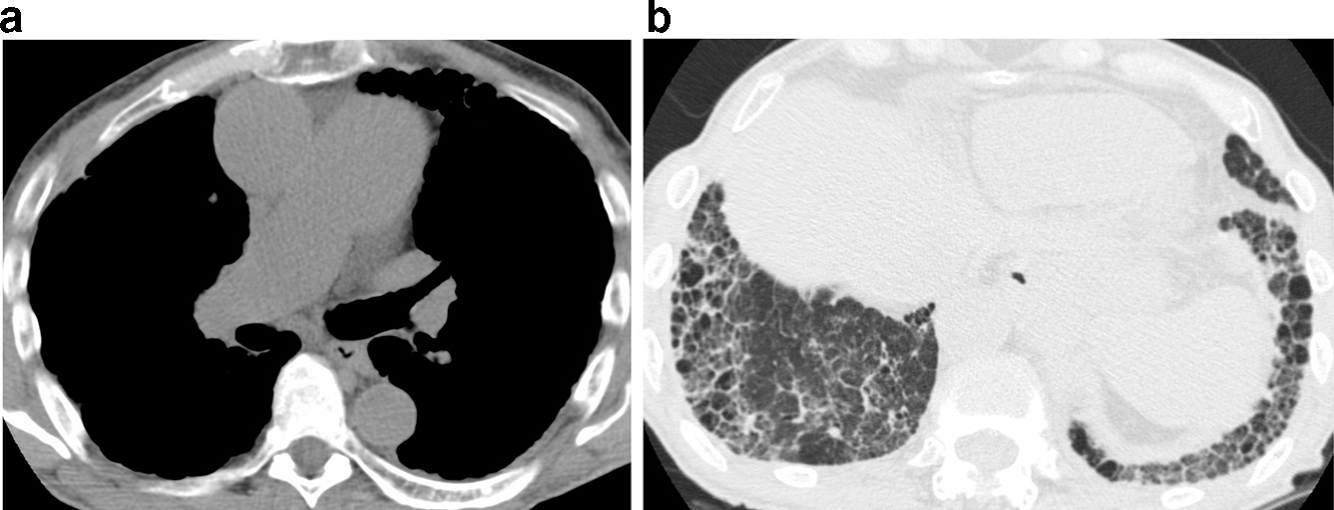
Marked dilation of the pulmonary artery (a) is evident in this patient with pulmonary hypertension. A UIP pattern of basal and subpleural predominant honeycombing, reticulation, and traction bronchiectasis (b) is likely the driving cause for the pulmonary hypertension. UIP, usual interstitial pneumonia.

Pulmonary hypertension (PH) is a common comorbidity in patients with IPF and is associated with worse outcomes in these patients.^55^ Screening with transthoracic echocardiography (TTE) to evaluate right ventricular systolic pressures is frequently used to assess for the presence of PH. Unfortunately however, in the setting of fibrotic lung disease the accuracy of TTE to assess PH is diminished.^56^ Alternatively, a radiologic measurement of the main pulmonary artery (PA) size on HRCT may be useful in the assessment of PH. Specifically, two potential methods of diagnosing PH that have been described include the measurement of the main PA diameter, or alternatively measuring the ratio of the PA diameter to that of the ascending aorta (AA). While these measurements are easy to obtain on HRCT, there are conflicting results on how reliable they are in predicting PH. An early study by Tan et al demonstrated that in patients with parenchymal lung disease (83% being ILD patients) a main PA diameter >or =29 mm and segmental artery to bronchus ratio >1.1 in 3 or 4 lobes had a 100% specificity for PH verified on right heart catheterization.^57^ This study also showed that patients with parenchymal lung disease, a main PA diameter ≥29 mm alone had an 84% specificity and 75% specificity for PH. In patients with scleroderma and mild to moderate fibrosis, a main PA diameter >30 mm yielded a sensitivity of 81.3% and specificity of 87.5%, however the correlation diminished with worsening lung function.^58^ In another study, a PA:AA ratio >1 was associated with lower transplant free survival in patients with IPF, however, there was only a weak correlation with PA:AA ratio and right heart catheterization diagnosed PH (Figure 13).^59^ Furthermore, it has been hypothesized that pulmonary fibrosis may lead to traction-related pulmonary artery dilation, and therefore PA diameter may be more of a marker of disease severity, which has lead to some hesitancy in using it to identify PH.

## Longitudinal evaluation

Progressive disease on imaging has been shown to have negative prognostic implications.^60–62^ Longitudinal evaluation may also identify complications such as lung cancer. The appropriate frequency of HRCT scans is unknown. We generally perform annual chest CT imaging to inform prognosis. HRCT is also indicated in patients with suspected acute respiratory declines. A detailed position paper is available on the use of CT staging and monitoring for ILD, as well as the potential role of quantitative CT techniques.^63^

## Conclusions

The radiologist is essential in the evaluation of suspected IPF, and thus must be knowledgeable of the updated HRCT classification scheme for UIP. IPF can be diagnosed with a typical UIP pattern on HRCT and the appropriate clinical context, avoiding the need for surgical lung biopsy. The role of surgical lung biopsy should be discussed in MDD for cases with imaging patterns other than typical UIP or in which the clinical context is not typical for IPF.
